# 3-[4-(10*H*-Indolo[3,2-*b*]quinolin-11-yl)piperazin-1-yl]propan-1-ol

**DOI:** 10.1107/S1600536811050215

**Published:** 2011-11-30

**Authors:** Gary S. Nichol, Peda V. L. Boddupally, Biswanath De, Laurence H. Hurley

**Affiliations:** a Department of Chemistry and Biochemistry, The University of Arizona, Tucson, AZ 85716, USA; b College of Pharmacy, The University of Arizona, Tucson, AZ 85721, USA

## Abstract

In the title compound, C_22_H_24_N_4_O, the aromatic moiety is essentially planar (r.m.s. deviation of a least-squares plane fitted through all non-H atoms = 0.0386 Å) and is rotated by 89.98 (4)° from the piperazine ring, which adopts the expected chair conformation. The propanol chain is not fully extended away from the piperazine ring. In the crystal, there are two unique hydrogen-bonding inter­actions. One is an O—H⋯N inter­action which, together with an inversion-related symmetry equivalent, forms a ring motif. The second is an N—H⋯N inter­action which links adjacent mol­ecules by means of a chain motif which propagates in the *c*-axis direction. Overall, a two-dimensional hydrogen-bonded structure is formed.

## Related literature

For background information on the synthesis and properties of quindolines, see: Guyen *et al.* (2004 ▶); Ou *et al.* (2007 ▶). For synthesis details, see: Bierer *et al.* (1998 ▶); Takeuchi *et al.* (1997 ▶). For the graph-set notation description of hydrogen bonding, see: Bernstein *et al.* (1995 ▶).
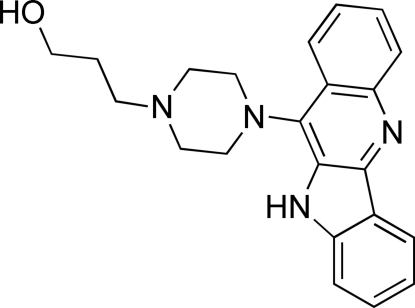


## Experimental

### 

#### Crystal data

C_22_H_24_N_4_O
*M* *_r_* = 360.45
Monoclinic, 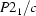

*a* = 11.218 (2) Å
*b* = 15.673 (3) Å
*c* = 11.847 (2) Å
β = 117.417 (2)°
*V* = 1849.0 (6) Å^3^
*Z* = 4
Mo *K*α radiation
μ = 0.08 mm^−1^
*T* = 120 K
0.51 × 0.36 × 0.25 mm

#### Data collection

Bruker SMART 1000 CCD diffractometer
Absorption correction: multi-scan (*SADABS*; Sheldrick, 1996 ▶) *T*_min_ = 0.929, *T*_max_ = 0.980
9503 measured reflections
3446 independent reflections
2678 reflections with *I* > 2σ(*I*)
*R*_int_ = 0.027

#### Refinement

*R*[*F* ^2^ > 2σ(*F* ^2^)] = 0.039
*wR*(*F* ^2^) = 0.108
*S* = 1.03
3446 reflections
340 parameters
All H-atom parameters refined
Δρ_max_ = 0.27 e Å^−3^
Δρ_min_ = −0.22 e Å^−3^

### 

Data collection: *SMART* (Bruker, 2007 ▶); cell refinement: *SAINT* (Bruker, 2007 ▶); data reduction: *SAINT*; program(s) used to solve structure: *SHELXTL* (Sheldrick, 2008 ▶); program(s) used to refine structure: *SHELXTL*; molecular graphics: *ORTEP-3 for Windows* (Farrugia, 1997 ▶) and *Mercury* (Macrae *et al.*, 2006 ▶); software used to prepare material for publication: *SHELXTL* and *publCIF* (Westrip, 2010 ▶).

## Supplementary Material

Crystal structure: contains datablock(s) I, global. DOI: 10.1107/S1600536811050215/fj2477sup1.cif

Supplementary material file. DOI: 10.1107/S1600536811050215/fj2477Isup2.cdx

Structure factors: contains datablock(s) I. DOI: 10.1107/S1600536811050215/fj2477Isup3.hkl

Supplementary material file. DOI: 10.1107/S1600536811050215/fj2477Isup4.cml

Additional supplementary materials:  crystallographic information; 3D view; checkCIF report

## Figures and Tables

**Table 1:** Hydrogen-bond geometry (Å, °)

*D*—H⋯*A*	*D*—H	H⋯*A*	*D*⋯*A*	*D*—H⋯*A*
O—H1*O*⋯N4^i^	0.97 (3)	1.94 (3)	2.8990 (18)	169 (2)
N1—H1*N*⋯N2^ii^	0.885 (19)	1.99 (2)	2.866 (2)	168.4 (17)

Symmetry codes: (i) 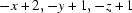; (ii) 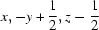.

**Fig. 1.**
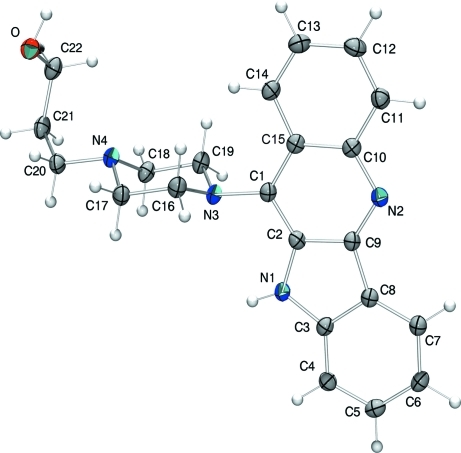
The molecular structure of (I), with anisotropic displacement ellipsoids at the 50% probability level.

**Fig. 2.**
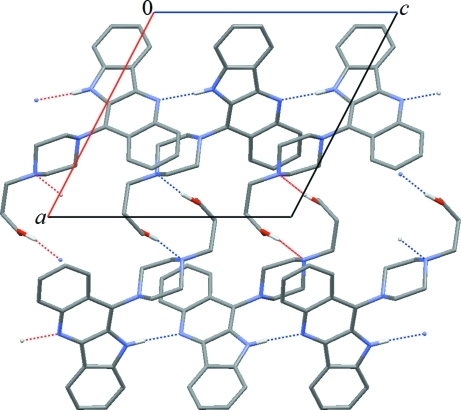
Hydrogen bonding interactions (blue dashed lines) in (I). Red dashed lines indicate continuation of hydrogen bonding. C-bound H atoms are omitted.

## supplementary crystallographic information

### Comment

3-(4-(10*H*-Indolo[3,2-*b*]quinolin-11-yl)piperazin-1-yl)propan-1-ol, (I), was synthesized as a potential c-Myc G-quadruplex interactive agent. Quindolines were first reported as telomeric G-quadruplex stabilizing agent by Guyen *et al.* (2004). Ou *et al.* (2007) synthesized and tested a series of 11-substituted quindoline analogs for G-quadruplex stabilization and c-Myc downregulation. We have used quindoline as a scaffold for lead modification and synthesis of c-Myc G-quadruplex stabilizing compounds. We postulated that the addition of piperazine ring would provide steric bulk to the planar quindoline ring resulting in increased selectivity for G-quadruplex binding over duplex DNA. The title compound was synthesized starting from 11-chloroquindoline and tested for its ability to interact with c-Myc G-quadruplex. Anthranilic acid and aniline were used in a multistep procedure to synthesize 11-chloroquindoline as reported in literature (Bierer *et al.*, 1998; Takeuchi *et al.*, 1997).

The molecular structure of (I) is shown in Figure 1. Molecular dimensions are unexceptional. The aromatic moiety of the structure is essentially planar (a mean plane fitted through all non-hydrogen atoms of the moiety has an r.m.s. deviation of 0.0386 Å). This plane is rotated by 89.98 (4)° from the piperazine ring, which adopts an expected chair conformation. The propanol chain is not fully extended away from the piperazine ring.

The compound has a two-dimensional hydrogen-bonded structure (Figure 2). Two O–H···N interactions, which are symmetry related by an inversion centre, form an *R*^2^_2_(12)motif (Bernstein *et al.*, 1995) while further N–H···N interactions link adjacent molecules into by means of a *C*(5) motif in the *c-*axis direction.

### Experimental

11-Chloroquindoline was synthesized according to a literature method (Bierer *et al.*, 1998; Takeuchi *et al.*, 1997). A mixture of 11-chloroquindoline (500 mg, 1.98 mmol) and 3-(piperazin-1-yl)propan-1-ol (1.5 ml) was heated at 100 ^0^C for 24 h, and the crude product on further purification gave 620 mg (86%) of the title compound, (I), as a yellow solid. Crystals for X-ray analysis were obtained by recrystallization from methanol: chloroform (4:1). ^1^H NMR (300 MHz, DMSO-*d*_6_): d 10.88 (br s, 1H, NH), 8.42–8.25 (m, 2H, ArH), 8.14 (d, J = 8.1 Hz, 1H, ArH), 7.76–7.50 (m, 4H, ArH), 7.25 (t, J = 6.4 Hz,1*H*, ArH), 5.10 - 4.30 (m, OH), 3.70–3.40 (m, 6H), 2.92–2.63 (m, 4H), 2.58–2.50 (m, 2H), 1.80–1.58 (m, 2H). ^13^C NMR (75 MHz, DMSO-*d*_6_): d 148.17, 145.80, 144.81, 136.73, 130.30, 129.89, 127.23, 127.02, 125.10, 124.27, 124.14, 122.09, 121.94, 120.20, 112.89, 60.29, 56.25, 54.53, 51.55, 30.54. MS (ESI): m/*z* = 361.2 [100%, (*M*+H)^+^]. HRMS calcd for C_22_H_25_N_4_O [*M*+H]^+^ 361.2023, found 361.2022. HPLC MS purity 100%.

### Refinement

All H atoms were located in a difference map and are freely refined.

### Crystal data

**Table:**

C_22_H_24_N_4_O	*F*(000) = 768
*M**_r_* = 360.45	*D*_x_ = 1.295 Mg m^−^^3^
Monoclinic, *P*2_1_/*c*	Mo *K*α radiation, λ = 0.71073 Å
Hall symbol: -P 2ybc	Cell parameters from 3837 reflections
*a* = 11.218 (2) Å	θ = 2.3–28.0°
*b* = 15.673 (3) Å	µ = 0.08 mm^−^^1^
*c* = 11.847 (2) Å	*T* = 120 K
β = 117.417 (2)°	Prism, dark brown
*V* = 1849.0 (6) Å^3^	0.51 × 0.36 × 0.25 mm
*Z* = 4	

### Data collection

**Table:**

Bruker SMART 1000 CCD diffractometer	3446 independent reflections
Radiation source: sealed tube	2678 reflections with *I* > 2σ(*I*)
graphite	*R*_int_ = 0.027
thin–slice ω scans	θ_max_ = 25.5°, θ_min_ = 2.1°
Absorption correction: multi-scan (*SADABS*; Sheldrick, 1996)	*h* = −12→13
*T*_min_ = 0.929, *T*_max_ = 0.980	*k* = −18→18
9503 measured reflections	*l* = −14→9

### Refinement

**Table:**

Refinement on *F*^2^	Primary atom site location: structure-invariant direct methods
Least-squares matrix: full	Secondary atom site location: difference Fourier map
*R*[*F*^2^ > 2σ(*F*^2^)] = 0.039	Hydrogen site location: difference Fourier map
*wR*(*F*^2^) = 0.108	All H-atom parameters refined
*S* = 1.03	*w* = 1/[σ^2^(*F*_o_^2^) + (0.0523*P*)^2^ + 0.7097*P*] where *P* = (*F*_o_^2^ + 2*F*_c_^2^)/3
3446 reflections	(Δ/σ)_max_ < 0.001
340 parameters	Δρ_max_ = 0.27 e Å^−^^3^
0 restraints	Δρ_min_ = −0.22 e Å^−^^3^

### Special details

**Table:**

Geometry. All e.s.d.'s (except the e.s.d. in the dihedral angle between two l.s. planes) are estimated using the full covariance matrix. The cell e.s.d.'s are taken into account individually in the estimation of e.s.d.'s in distances, angles and torsion angles; correlations between e.s.d.'s in cell parameters are only used when they are defined by crystal symmetry. An approximate (isotropic) treatment of cell e.s.d.'s is used for estimating e.s.d.'s involving l.s. planes.
Refinement. Refinement of *F*^2^ against ALL reflections. The weighted *R*-factor *wR* and goodness of fit *S* are based on *F*^2^, conventional *R*-factors *R* are based on *F*, with *F* set to zero for negative *F*^2^. The threshold expression of *F*^2^ > σ(*F*^2^) is used only for calculating *R*-factors(gt) *etc*. and is not relevant to the choice of reflections for refinement. *R*-factors based on *F*^2^ are statistically about twice as large as those based on *F*, and *R*- factors based on ALL data will be even larger.

### Fractional atomic coordinates and isotropic or equivalent isotropic displacement parameters (Å^2^)

**Table:**

	*x*	*y*	*z*	*U*_iso_*/*U*_eq_	
O	1.06789 (12)	0.50790 (7)	0.41013 (11)	0.0267 (3)	
H1O	1.109 (2)	0.5355 (16)	0.493 (2)	0.068 (8)*	
N1	0.37444 (13)	0.24064 (9)	0.40400 (13)	0.0220 (3)	
H1N	0.3956 (18)	0.2294 (11)	0.3422 (18)	0.028 (5)*	
N2	0.42965 (13)	0.31976 (8)	0.70450 (12)	0.0208 (3)	
N3	0.61399 (13)	0.34403 (8)	0.45703 (12)	0.0209 (3)	
N4	0.79071 (12)	0.39539 (8)	0.35679 (12)	0.0210 (3)	
C1	0.55854 (15)	0.33858 (10)	0.54422 (15)	0.0198 (3)	
C2	0.44663 (15)	0.28845 (10)	0.51126 (15)	0.0198 (3)	
C3	0.26853 (15)	0.20239 (10)	0.41345 (15)	0.0207 (3)	
C4	0.16905 (16)	0.14933 (10)	0.32651 (16)	0.0227 (4)	
H4	0.1712 (16)	0.1326 (11)	0.2482 (17)	0.024 (4)*	
C5	0.06949 (16)	0.12188 (11)	0.35618 (16)	0.0242 (4)	
H5	0.0003 (18)	0.0838 (12)	0.2954 (17)	0.028 (5)*	
C6	0.06798 (16)	0.14638 (11)	0.46923 (16)	0.0239 (4)	
H6	−0.0062 (17)	0.1263 (11)	0.4887 (16)	0.027 (5)*	
C7	0.16832 (16)	0.19778 (10)	0.55680 (16)	0.0221 (4)	
H7	0.1694 (16)	0.2136 (10)	0.6364 (16)	0.020 (4)*	
C8	0.26991 (15)	0.22583 (10)	0.52887 (15)	0.0199 (3)	
C9	0.38517 (15)	0.28109 (10)	0.59321 (15)	0.0193 (3)	
C10	0.54068 (15)	0.37040 (10)	0.74025 (15)	0.0202 (3)	
C11	0.58953 (16)	0.41338 (11)	0.85823 (15)	0.0249 (4)	
H11	0.5411 (16)	0.4042 (10)	0.9084 (15)	0.016 (4)*	
C12	0.69713 (17)	0.46688 (11)	0.89926 (16)	0.0275 (4)	
H12	0.7267 (16)	0.4987 (11)	0.9796 (17)	0.023 (4)*	
C13	0.76274 (17)	0.47893 (11)	0.82389 (17)	0.0264 (4)	
H13	0.839 (2)	0.5184 (13)	0.8534 (19)	0.038 (5)*	
C14	0.72030 (16)	0.43782 (10)	0.70995 (16)	0.0226 (4)	
H14	0.7664 (17)	0.4464 (11)	0.6598 (16)	0.026 (5)*	
C15	0.60766 (15)	0.38205 (10)	0.66306 (15)	0.0199 (3)	
C16	0.60048 (17)	0.42857 (11)	0.39894 (16)	0.0240 (4)	
H16A	0.6495 (17)	0.4746 (11)	0.4637 (16)	0.023 (4)*	
H16B	0.5062 (19)	0.4439 (11)	0.3571 (17)	0.028 (5)*	
C17	0.65126 (16)	0.42647 (12)	0.30064 (16)	0.0244 (4)	
H17A	0.6487 (17)	0.4852 (12)	0.2682 (16)	0.025 (5)*	
H17B	0.5923 (17)	0.3887 (11)	0.2266 (16)	0.024 (4)*	
C18	0.79162 (17)	0.30740 (10)	0.39967 (17)	0.0237 (4)	
H18B	0.8840 (17)	0.2845 (11)	0.4334 (16)	0.021 (4)*	
H18A	0.7292 (19)	0.2708 (12)	0.3271 (18)	0.034 (5)*	
C19	0.74808 (17)	0.30570 (11)	0.50322 (16)	0.0231 (4)	
H19A	0.8171 (18)	0.3353 (11)	0.5797 (17)	0.028 (5)*	
H19B	0.7437 (17)	0.2446 (12)	0.5249 (16)	0.023 (4)*	
C20	0.83818 (17)	0.40109 (11)	0.26025 (16)	0.0251 (4)	
H20A	0.7803 (17)	0.3667 (11)	0.1848 (17)	0.023 (4)*	
H20B	0.8255 (16)	0.4632 (11)	0.2318 (15)	0.017 (4)*	
C21	0.98381 (17)	0.37398 (11)	0.30578 (17)	0.0274 (4)	
H21A	1.0040 (17)	0.3868 (12)	0.2342 (17)	0.030 (5)*	
H21B	0.9945 (17)	0.3105 (12)	0.3171 (17)	0.029 (5)*	
C22	1.08386 (17)	0.41778 (11)	0.42631 (17)	0.0266 (4)	
H22A	1.1778 (18)	0.4023 (11)	0.4449 (16)	0.024 (4)*	
H22B	1.0721 (16)	0.3979 (11)	0.5050 (16)	0.023 (4)*	

### Atomic displacement parameters (Å^2^)

**Table:**

	*U*^11^	*U*^22^	*U*^33^	*U*^12^	*U*^13^	*U*^23^
O	0.0287 (6)	0.0227 (6)	0.0286 (7)	−0.0009 (5)	0.0130 (5)	0.0010 (5)
N1	0.0230 (7)	0.0257 (8)	0.0216 (7)	−0.0035 (6)	0.0140 (6)	−0.0041 (6)
N2	0.0214 (7)	0.0218 (7)	0.0204 (7)	0.0013 (5)	0.0105 (6)	0.0006 (6)
N3	0.0204 (7)	0.0222 (7)	0.0239 (7)	0.0000 (5)	0.0135 (6)	0.0012 (6)
N4	0.0209 (7)	0.0208 (7)	0.0244 (7)	0.0006 (5)	0.0131 (6)	0.0014 (5)
C1	0.0193 (8)	0.0200 (8)	0.0213 (8)	0.0031 (6)	0.0104 (6)	0.0024 (6)
C2	0.0200 (8)	0.0198 (8)	0.0195 (8)	0.0021 (6)	0.0091 (7)	−0.0004 (6)
C3	0.0194 (8)	0.0198 (8)	0.0237 (8)	0.0014 (6)	0.0106 (7)	0.0019 (6)
C4	0.0242 (8)	0.0222 (8)	0.0216 (8)	0.0006 (7)	0.0106 (7)	−0.0010 (7)
C5	0.0199 (8)	0.0212 (8)	0.0275 (9)	−0.0010 (7)	0.0074 (7)	0.0011 (7)
C6	0.0196 (8)	0.0234 (9)	0.0305 (9)	0.0000 (7)	0.0131 (7)	0.0030 (7)
C7	0.0222 (8)	0.0225 (9)	0.0232 (8)	0.0012 (6)	0.0118 (7)	0.0017 (7)
C8	0.0201 (8)	0.0188 (8)	0.0213 (8)	0.0020 (6)	0.0099 (7)	0.0017 (6)
C9	0.0197 (8)	0.0186 (8)	0.0210 (8)	0.0020 (6)	0.0105 (7)	0.0010 (6)
C10	0.0191 (8)	0.0181 (8)	0.0226 (8)	0.0031 (6)	0.0089 (7)	0.0019 (6)
C11	0.0252 (8)	0.0268 (9)	0.0243 (9)	0.0009 (7)	0.0127 (7)	−0.0005 (7)
C12	0.0273 (9)	0.0281 (9)	0.0239 (9)	−0.0009 (7)	0.0090 (7)	−0.0067 (7)
C13	0.0208 (8)	0.0259 (9)	0.0297 (9)	−0.0020 (7)	0.0092 (7)	−0.0034 (7)
C14	0.0194 (8)	0.0221 (9)	0.0271 (9)	0.0018 (6)	0.0115 (7)	0.0003 (7)
C15	0.0184 (8)	0.0183 (8)	0.0228 (8)	0.0042 (6)	0.0093 (7)	0.0024 (6)
C16	0.0204 (8)	0.0255 (9)	0.0266 (9)	0.0041 (7)	0.0113 (7)	0.0056 (7)
C17	0.0233 (9)	0.0271 (9)	0.0243 (9)	0.0012 (7)	0.0122 (7)	0.0049 (7)
C18	0.0255 (9)	0.0199 (8)	0.0314 (9)	−0.0002 (7)	0.0178 (8)	−0.0014 (7)
C19	0.0240 (8)	0.0193 (9)	0.0304 (9)	0.0032 (7)	0.0164 (8)	0.0040 (7)
C20	0.0295 (9)	0.0270 (10)	0.0235 (9)	−0.0034 (7)	0.0162 (8)	−0.0020 (7)
C21	0.0331 (10)	0.0247 (9)	0.0341 (10)	−0.0005 (7)	0.0239 (8)	−0.0013 (8)
C22	0.0260 (9)	0.0223 (9)	0.0357 (10)	0.0026 (7)	0.0178 (8)	0.0052 (7)

### Geometric parameters (Å, °)

**Table:**

O—H1O	0.97 (3)	C10—C15	1.438 (2)
O—C22	1.426 (2)	C11—H11	0.984 (16)
N1—H1N	0.885 (19)	C11—C12	1.362 (2)
N1—C2	1.373 (2)	C12—H12	0.987 (18)
N1—C3	1.381 (2)	C12—C13	1.407 (2)
N2—C9	1.322 (2)	C13—H13	0.98 (2)
N2—C10	1.369 (2)	C13—C14	1.368 (2)
N3—C1	1.433 (2)	C14—H14	0.960 (18)
N3—C16	1.468 (2)	C14—C15	1.422 (2)
N3—C19	1.471 (2)	C16—H16A	1.011 (18)
N4—C17	1.473 (2)	C16—H16B	0.969 (19)
N4—C18	1.468 (2)	C16—C17	1.514 (2)
N4—C20	1.470 (2)	C17—H17A	0.992 (18)
C1—C2	1.376 (2)	C17—H17B	1.011 (17)
C1—C15	1.427 (2)	C18—H18B	0.991 (17)
C2—C9	1.431 (2)	C18—H18A	1.00 (2)
C3—C4	1.393 (2)	C18—C19	1.515 (2)
C3—C8	1.409 (2)	C19—H19A	0.995 (18)
C4—H4	0.976 (17)	C19—H19B	0.999 (18)
C4—C5	1.385 (2)	C20—H20A	0.988 (18)
C5—H5	0.981 (18)	C20—H20B	1.019 (17)
C5—C6	1.401 (2)	C20—C21	1.526 (2)
C6—H6	1.011 (18)	C21—H21A	0.994 (19)
C6—C7	1.385 (2)	C21—H21B	1.004 (19)
C7—H7	0.969 (17)	C21—C22	1.515 (2)
C7—C8	1.396 (2)	C22—H22A	1.002 (18)
C8—C9	1.448 (2)	C22—H22B	1.048 (17)
C10—C11	1.415 (2)		
			
H1O—O—C22	109.5 (14)	H13—C13—C14	120.0 (12)
H1N—N1—C2	127.3 (12)	C13—C14—H14	120.2 (11)
H1N—N1—C3	123.3 (12)	C13—C14—C15	121.17 (16)
C2—N1—C3	108.97 (13)	H14—C14—C15	118.6 (10)
C9—N2—C10	116.48 (13)	C1—C15—C10	119.30 (14)
C1—N3—C16	113.97 (12)	C1—C15—C14	123.29 (14)
C1—N3—C19	114.61 (12)	C10—C15—C14	117.40 (14)
C16—N3—C19	114.20 (12)	N3—C16—H16A	112.7 (10)
C17—N4—C18	107.68 (13)	N3—C16—H16B	108.4 (11)
C17—N4—C20	108.57 (12)	N3—C16—C17	110.28 (14)
C18—N4—C20	112.26 (13)	H16A—C16—H16B	107.2 (14)
N3—C1—C2	118.14 (14)	H16A—C16—C17	109.4 (10)
N3—C1—C15	125.75 (14)	H16B—C16—C17	108.7 (10)
C2—C1—C15	116.10 (14)	N4—C17—C16	110.94 (13)
N1—C2—C1	130.28 (15)	N4—C17—H17A	108.5 (10)
N1—C2—C9	108.71 (13)	N4—C17—H17B	109.4 (9)
C1—C2—C9	121.00 (14)	C16—C17—H17A	108.9 (10)
N1—C3—C4	128.75 (15)	C16—C17—H17B	110.6 (10)
N1—C3—C8	109.88 (14)	H17A—C17—H17B	108.4 (14)
C4—C3—C8	121.36 (15)	N4—C18—H18B	108.6 (10)
C3—C4—H4	120.1 (10)	N4—C18—H18A	110.5 (11)
C3—C4—C5	117.59 (15)	N4—C18—C19	110.06 (13)
H4—C4—C5	122.3 (10)	H18B—C18—H18A	109.1 (14)
C4—C5—H5	117.4 (10)	H18B—C18—C19	109.5 (10)
C4—C5—C6	121.58 (15)	H18A—C18—C19	109.0 (11)
H5—C5—C6	121.0 (10)	N3—C19—C18	110.32 (14)
C5—C6—H6	120.4 (10)	N3—C19—H19A	112.5 (10)
C5—C6—C7	120.82 (15)	N3—C19—H19B	108.9 (10)
H6—C6—C7	118.7 (10)	C18—C19—H19A	108.9 (10)
C6—C7—H7	121.3 (10)	C18—C19—H19B	107.3 (10)
C6—C7—C8	118.45 (15)	H19A—C19—H19B	108.8 (14)
H7—C7—C8	120.3 (10)	N4—C20—H20A	110.4 (10)
C3—C8—C7	120.17 (14)	N4—C20—H20B	105.7 (9)
C3—C8—C9	105.96 (13)	N4—C20—C21	114.87 (14)
C7—C8—C9	133.82 (15)	H20A—C20—H20B	106.8 (13)
N2—C9—C2	124.13 (14)	H20A—C20—C21	108.6 (10)
N2—C9—C8	129.39 (14)	H20B—C20—C21	110.3 (9)
C2—C9—C8	106.48 (13)	C20—C21—H21A	105.6 (10)
N2—C10—C11	117.70 (14)	C20—C21—H21B	111.4 (10)
N2—C10—C15	122.98 (14)	C20—C21—C22	114.52 (14)
C11—C10—C15	119.32 (14)	H21A—C21—H21B	104.8 (14)
C10—C11—H11	117.2 (9)	H21A—C21—C22	110.6 (10)
C10—C11—C12	121.46 (16)	H21B—C21—C22	109.4 (10)
H11—C11—C12	121.3 (9)	O—C22—C21	109.25 (14)
C11—C12—H12	120.3 (10)	O—C22—H22A	108.9 (10)
C11—C12—C13	119.52 (16)	O—C22—H22B	110.8 (9)
H12—C12—C13	120.1 (10)	C21—C22—H22A	110.2 (10)
C12—C13—H13	118.9 (12)	C21—C22—H22B	111.2 (9)
C12—C13—C14	121.11 (16)	H22A—C22—H22B	106.4 (13)
			
C16—N3—C1—C2	112.37 (16)	C7—C8—C9—C2	−176.76 (17)
C16—N3—C1—C15	−66.45 (19)	C9—N2—C10—C11	179.26 (14)
C19—N3—C1—C2	−113.44 (16)	C9—N2—C10—C15	−0.1 (2)
C19—N3—C1—C15	67.74 (19)	N2—C10—C11—C12	−178.11 (15)
C3—N1—C2—C1	−178.98 (16)	C15—C10—C11—C12	1.3 (2)
C3—N1—C2—C9	−0.12 (17)	C10—C11—C12—C13	−1.0 (3)
N3—C1—C2—N1	−0.4 (2)	C11—C12—C13—C14	0.0 (3)
N3—C1—C2—C9	−179.17 (13)	C12—C13—C14—C15	0.6 (3)
C15—C1—C2—N1	178.51 (15)	C13—C14—C15—C1	179.20 (15)
C15—C1—C2—C9	−0.2 (2)	C13—C14—C15—C10	−0.2 (2)
C2—N1—C3—C4	179.23 (16)	N3—C1—C15—C10	179.73 (14)
C2—N1—C3—C8	0.45 (18)	N3—C1—C15—C14	0.3 (2)
N1—C3—C4—C5	−177.20 (15)	C2—C1—C15—C10	0.9 (2)
C8—C3—C4—C5	1.5 (2)	C2—C1—C15—C14	−178.53 (14)
C3—C4—C5—C6	0.1 (2)	N2—C10—C15—C1	−0.8 (2)
C4—C5—C6—C7	−1.4 (2)	N2—C10—C15—C14	178.69 (14)
C5—C6—C7—C8	1.1 (2)	C11—C10—C15—C1	179.88 (14)
C6—C7—C8—C3	0.5 (2)	C11—C10—C15—C14	−0.7 (2)
C6—C7—C8—C9	177.43 (16)	C1—N3—C16—C17	−175.37 (13)
N1—C3—C8—C7	177.12 (14)	C19—N3—C16—C17	50.25 (18)
N1—C3—C8—C9	−0.60 (17)	C18—N4—C17—C16	62.64 (17)
C4—C3—C8—C7	−1.8 (2)	C20—N4—C17—C16	−175.58 (14)
C4—C3—C8—C9	−179.48 (14)	N3—C16—C17—N4	−55.76 (18)
C10—N2—C9—C2	0.8 (2)	C17—N4—C18—C19	−63.16 (17)
C10—N2—C9—C8	−178.45 (15)	C20—N4—C18—C19	177.38 (13)
N1—C2—C9—N2	−179.67 (14)	C1—N3—C19—C18	174.66 (13)
N1—C2—C9—C8	−0.25 (17)	C16—N3—C19—C18	−51.25 (18)
C1—C2—C9—N2	−0.7 (2)	N4—C18—C19—N3	57.42 (18)
C1—C2—C9—C8	178.74 (14)	C17—N4—C20—C21	177.89 (14)
C3—C8—C9—N2	179.89 (15)	C18—N4—C20—C21	−63.17 (18)
C3—C8—C9—C2	0.51 (17)	N4—C20—C21—C22	−52.4 (2)
C7—C8—C9—N2	2.6 (3)	C20—C21—C22—O	−53.75 (19)

### Hydrogen-bond geometry (Å, °)

**Table:**

*D*—H···*A*	*D*—H	H···*A*	*D*···*A*	*D*—H···*A*
O—H1O···N4^i^	0.97 (3)	1.94 (3)	2.8990 (18)	169 (2)
N1—H1N···N2^ii^	0.885 (19)	1.99 (2)	2.866 (2)	168.4 (17)

Symmetry codes: (i) −*x*+2, −*y*+1, −*z*+1; (ii) *x*, −*y*+1/2, *z*−1/2.
